# Calmodulin-Binding Proteins in Muscle: A Minireview on Nuclear Receptor Interacting Protein, Neurogranin, and Growth-Associated Protein 43

**DOI:** 10.3390/ijms21031016

**Published:** 2020-02-04

**Authors:** Fereshteh Moradi, Emily N. Copeland, Ryan W. Baranowski, Aiden E. Scholey, Jeffrey A. Stuart, Val A. Fajardo

**Affiliations:** 1 Department of Biological Sciences, Brock University, St. Catharines, ON L2S 3A1, Canada; fm15ta@brocku.ca (F.M.); jstuart@brocku.ca (J.A.S.); 2 Centre for Neuroscience, Brock University, St. Catharines, ON L2S 3A1, Canada; ec15wf@brocku.ca; 3 Centre for Bone and Muscle Health, Brock University, St. Catharines, ON L2S 3A1, Canada; rb15xz@brocku.ca; 4 Department of Kinesiology, Brock University, St. Catharines, ON L2S 3A1, Canada; as15js@brocku.ca

**Keywords:** calcineurin, CaMKII, neurogranin, GAP43, neuromodulin, NRIP, IQ-motif

## Abstract

Calmodulin (CaM) is an important Ca^2+^-sensing protein with numerous downstream targets that are either CaM-dependant or CaM-regulated. In muscle, CaM-dependent proteins, which are critical regulators of dynamic Ca^2+^ handling and contractility, include calcineurin (CaN), CaM-dependant kinase II (CaMKII), ryanodine receptor (RyR), and dihydropyridine receptor (DHPR). CaM-regulated targets include genes associated with oxidative metabolism, muscle plasticity, and repair. Despite its importance in muscle, the regulation of CaM—particularly its availability to bind to and activate downstream targets—is an emerging area of research. In this minireview, we discuss recent studies revealing the importance of small IQ motif proteins that bind to CaM to either facilitate (nuclear receptor interacting protein; NRIP) its activation of downstream targets, or sequester (neurogranin, Ng; and growth-associated protein 43, GAP43) CaM away from their downstream targets. Specifically, we discuss recent studies that have begun uncovering the physiological roles of NRIP, Ng, and GAP43 in skeletal and cardiac muscle, thereby highlighting the importance of endogenously expressed CaM-binding proteins and their regulation of CaM in muscle.

## 1. Introduction

Composed of two canonical EF-hand lobes, calmodulin (CaM) binds up to four Ca^2+^ ions. Upon Ca^2+^ binding, the EF-hand motifs undergo a conformational alteration, exposing hydrophobic sidechains to the surface and thereby engaging in hydrophobic protein–protein interactions with numerous downstream targets that include enzymes and ion channels [1]. In muscle, CaM can bind to and activate several proteins that regulate gene expression and Ca^2+^ handling, ultimately influencing muscle contractility, metabolism, and plasticity [2,3]. These include but are not limited to CaM-dependant kinase II (CaMKII), calcineurin (CaN), ryanodine receptor (RyR), and dihydropyridine receptor (DHPR) [3,4,5,6,7]. Despite its established importance in muscle, the regulation of CaM—specifically its ability to bind to and activate its downstream targets—is emerging as an important area of research. 

The IQ motif is a loosely conserved domain that begins with isoleucine (I) and glutamine (Q) and generally consists of IQXXXRGXXXR, where the X represents non-conserved residues. With over 900 Pfam entries, the IQ motif is widely distributed, and for a more comprehensive review, readers are referred to Bahler et al. [8]. Proteins that contain the IQ motif are able to bind to CaM in its Ca^2+^-free (apo-CaM) and/or Ca^2+^-bound states. Therefore, these IQ motif proteins may regulate CaM availability at low and high Ca^2+^ levels by sequestering CaM away from its downstream targets, or facilitating its interaction with other proteins by concentrating CaM in specifically localized pools. In this minireview, we discuss recent research revealing the physiological roles of three small IQ motif proteins in skeletal and cardiac muscle: nuclear receptor interacting protein (NRIP), growth-associated protein 43 (GAP43), and neurogranin (Ng).

## 2. Nuclear Receptor Interacting Protein

NRIP is an androgen receptor (AR)-interacting protein that has an IQ motif enabling CaM binding [9]. Its interaction with CaM is Ca^2+^ dependent, as CaM binding is diminished in the presence of the Ca^2+^ chelator EGTA [10]. NRIP is highly expressed in cardiac and skeletal muscle where it is specifically localized at the Z-disc [9,11]. In skeletal muscle, NRIP co-localizes with CaN, which is a Ca^2+^/CaM-dependant phosphatase that regulates gene expression by dephosphorylating nuclear factor of activated T cells (NFAT), leading to their subsequent nuclear entry. NFAT is a multigene family of inducible nuclear transcription factors containing five members: NFATc1, NFATc2, NFATc3, NFATc4, and NFAT5 [12]. All NFAT family members, except NFAT5, are regulated by CaN. Recently, Chen et al. [9] generated *Nrip*-knockout mice and demonstrated that, in the absence of NRIP, NFAT phosphorylation was enhanced, thereby revealing the importance of NRIP for calcineurin activation in rodent skeletal muscle. In addition, CaMKII is a Ca^2+^/CaM-dependent serine/threonine protein kinase that, upon CaM binding, can exhibit autonomous enzyme activity via intramolecular autophosphorylation [7]. Chen et al. found that CaMKII phosphorylation was reduced, suggesting that both CaN and CaMKII activation were impaired in the absence of NRIP. To explain this finding, co-immunoprecipitation experiments demonstrated reduced CaM–CaN and CaM–CaMKII binding in muscles from *Nrip*-null mice compared with the wild-type (WT), which highlights the role of NRIP in facilitating CaM binding and activation of CaN and CaMKII [9].

Together, CaMKII and CaN can work synergistically to increase the expression of genes associated with oxidative metabolism. CaMKII acts to increase myocyte enhancer factor 2 (MEF2) nuclear activity by phosphorylating its repressor, the class-II histone deacetylases (HDAC; -4, -5, and 7), leading to its subsequent export out of the nucleus [13,14,15]. In addition, CaMKII activity is also required for MEF2 binding to its target genes in skeletal muscle [16]. Along with dephosphorylated NFAT (via CaN), activated MEF2 (via CaMKII) promotes the expression of genes largely expressed in oxidative muscle fibre types, such as myosin heavy chain I, myoglobin, slow troponin, and peroxisome proliferator-activated receptor gamma coactivator 1-alpha (PGC1-α) [7,17,18]. Not surprisingly, Chen et al. showed that muscles from *Nrip*-null mice had a reduction in the proportion of slow-oxidative fibres and a concomitant increase in the fast-glycolytic fibres. Associated with this finding, the authors also showed that fatigue resistance was significantly impaired in *Nrip*-knockout isolated muscles and also in *Nrip*-knockout mice subjected to exhaustive treadmill running [9].

While an overall reduction in the oxidative phenotype can contribute to increased muscle fatigability, Chen et al. [9] also reported reductions in total sarcoplasmic reticulum (SR) Ca^2+^ content and release, without any changes in the expression of the RyR Ca^2+^ channels or the sarco(endo)plasmic reticulum Ca^2+^-ATPase (SERCA) pump. A reduction in releasable Ca^2+^ also translates into reduced force production and fatigue resistance in the *Nrip*-knockout mice. During normal excitation contraction coupling (ECC) in skeletal muscle, membrane depolarization sensed by the DHPR leads to RyR opening and Ca^2+^ release via a physical connection between DHPR and RyR. Both DHPR and RyR are regulated by CaM binding; however, modulation of skeletal muscle ECC via CaM is complex. Previous studies have shown that Ca^2+^/CaM binding to the IQ motif of skeletal muscle DHPR is involved in Ca^2+^-dependent inactivation of DHPR Ca^2+^ currents [19,20]. In contrast, mutations in the IQ motif of DHPR reduces Ca^2+^ currents [21]. Further, under low Ca^2+^ conditions, apo-CaM binds to RyR and acts to increase RyR channel openness, whereas under high Ca^2+^ conditions, Ca^2+^-CaM binds to RyR and partially lowers RyR open probability [22,23]. Altogether, these results suggest that CaM binding to DHPR and RyR could have multiple roles in modulating Ca^2+^ release. Given the role of NRIP in facilitating CaM binding with its downstream targets, the reductions in SR Ca^2+^ release observed with genetic *Nrip* deletion [9] may highlight a role for NRIP in stimulating Ca^2+^ release via CaM binding with DHPR and/or RyR. It is also possible that NRIP’s activation of CaMKII is critical for Ca^2+^ release, since CaMKII can phosphorylate RyR1 to increase its open probability [24]. A reduction in CaMKII activation may also contribute to reduced total SR Ca^2+^ content, since it is well known that CaMKII can phosphorylate and inactivate phospholamban (PLN)—a negative regulator of the SERCA pump [25]. Thus, in the absence of altered SERCA and RyR protein levels, as reported by Chen et al. [9], *Nrip*-null mice may exhibit lowered SERCA pump activation and RyR channel open probability leading to reduced SR total Ca^2+^ content and release.

With respect to muscle disease, NRIP expression was previously found to be lowered in the affected muscles of patients living with limb girdle muscular dystrophy [26]. Given the importance of NRIP in CaN and CaMKII activation and overall muscle contractility and fatigability, it is plausible that this downregulation of NRIP represents a maladaptive response in limb girdle muscular dystrophy. Indeed, Chen et al. [9] demonstrated that silencing *Nrip* expression in C2C12 cells attenuated myoblast fusion and differentiation, and that *Nrip*-null mice had delayed muscle regeneration after cardiotoxin injection. This is not surprising since CaN has long been linked with myogenic differentiation and fusion *in vitro* and muscle regeneration *in vivo* by stimulating the expression of proteins such as myogenin, interleukin-4, and stabilin-2 [27,28,29,30,31,32,33,34]. The latter is a phosphatidylserine receptor that plays an important role in promoting myoblast fusion and is regulated by CaN/NFAT signalling [29].

In cardiac muscle, recent evidence suggests that NRIP has a role in maintaining normal myocardial function, since muscle-specific *Nrip-null* mice displayed impaired contractility with a lowered left ventricular (LV) ejection fraction [11]. Yang et al. further demonstrated that isolated cardiomyocytes from *Nrip-*null mice had reduced rates of shortening and re-lengthening, which were associated with significant reductions in the peak Ca^2+^ amplitude and rate of Ca^2+^ decay. While these findings reveal the importance of NRIP in both systolic and diastolic function, no alterations in CaMKII nor NFAT phosphorylation were observed, and therefore unlike skeletal muscle, the impairments in cardiac contractility were not associated with changes in CaMKII or CaN activation. Rather, the cardiac muscle weakness and Ca^2+^ dysregulation were linked to an impairment in mitochondrial respiration and increase in mitochondrial reactive oxygen species (ROS) production. Yang et al. found that scavenging mitochondrial ROS with mitoTEMPO restored cardiac contractile function in the *Nrip-*null mice. Further, the *Nrip*-null mice also exhibited a cardiomyopathic phenotype that likely contributed to the impairments in cardiac contractility, including cardiac hypertrophy, ventricular dilation, aberrant sarcomeric (i.e., widening of Z-disk and narrowing of the I-band) and mitochondrial (i.e., cristae disarrangement) structure. Finally, Yang et al. reported significant reductions in NRIP expression in human and rodent failing hearts, which indicates that NRIP is not only important for normal cardiac function but could serve as a potential therapeutic target for heart failure.

## 3. Neurogranin

Neurogranin (Ng) is a small IQ motif containing protein that is particularly enriched in neuronal cells in the cerebral cortex, hippocampus, and striatum, where it is thought to either bind to and sequester CaM away from its downstream targets [35,36], or concentrate CaM and facilitate CaM activation of downstream targets in specialized localizations [37,38]. Ng is capable of binding to apo-CaM and Ca^2+-^CaM; when bound to CaM, Ng reduces its affinity for Ca^2+^ while also enhancing the rate of Ca^2+^ dissociation [39,40]. Together, these findings would suggest that Ng has a negative role in CaM activation. Ng was once thought to be a neuronal specific protein; however, we have recently discovered that Ng is expressed in mammalian skeletal muscle (rodent and human), and is particularly abundant in slow-oxidative muscle fibers [28]. Using C2C12 cells, our results showed that silencing *Ng* increased CaM-CaN binding—indicative of a role for Ng in sequestering CaM in these muscle cells. We also observed a significant reduction in NFAT phosphorylation and a significant increase in utrophin expression, which is a cytoskeletal protein controlled by CaN signalling [41]. Associated with enhanced CaN signalling, knocking down Ng also led to a significant enhancement of myoblast fusion and myogenic differentiation [28]. Given the role of CaN in muscle regeneration [27,28,29,30,31,32,33,34], it will be important to determine whether reducing Ng expression and increasing CaM–CaN binding in mice may enhance muscle regeneration *in vivo*. To date, the regulation of Ng on CaMKII signalling has not been investigated. In contrast with CaN, evidence in neuronal cells indicates that Ng concentrates CaM in dendritic spines, thereby activating CaMKII and long-term potentiation [42]. Thus, it will be important to determine whether Ng activates or inhibits CaMKII in rodent skeletal muscle. Finally, the enrichment of Ng in slow-oxidative muscle fibres [28] warrants the investigation of Ng and its physiological role in cardiac muscle where it may regulate CaN, CaMKII, and Ca^2+^ handling.

## 4. Growth-Associated Protein 43

GAP43, also known as neuromodulin, was discovered 30 years ago in synaptosomal plasma membranes of rat brain and is now known for its role in neuronal development and regeneration [43]. Similar to Ng, GAP43 contains an IQ domain that facilitates binding to CaM and is capable of binding to apo-CaM and Ca^2+^-CaM. GAP43 acts as a CaM “sponge” that binds to CaM and sequesters it away from its downstream targets [43]. Once believed to be a neuron-specific protein, recent evidence suggests that GAP43 is expressed in skeletal muscle [43,44,45], where it regulates dynamic handling of intracellular Ca^2+^. In 2016, Caprara et al. [45] examined the physiological role of GAP43 in skeletal muscle with *Gap43*-knockout mice. These mice showed low survival rates beyond weaning, a reduced adult body weight, decreased muscle strength, and an altered myofiber ultrastructure with no significant changes in the markers of satellite cell activation and myogenesis. Consistent with its putative role in dynamic Ca^2+^ regulation, myotubes isolated from surviving *Gap43*-null mice displayed an increased amplitude of Ca^2+^ release in response to depolarization (KCl treatment) and caffeine. The emerging hypothesis from Caprara et al. [45] was that GAP43 regulates downstream CaM interactions with RyR and DHPR to modulate Ca^2+^ channel opening; that is, in the absence of GAP43, CaM readily binds to RyR and DHPR leading to their activation. Indeed, treating the *Gap43*-null myotubes with W7 CaM inhibitor reduced the Ca^2+^ amplitude [45]. Though neither CaN or CaMKII activation have been directly examined in muscles from *Gap43*-null mice, Caprara et al. [45] did not observe any effects of *Gap43* deletion (heterozygous or homozygous) on myoblast fusion and differentiation in primary myoblasts. Given the role of CaN in regulating myoblast fusion and myogenic differentiation [27,28], these results suggest that GAP43 may have less of a role in regulating CaN and is rather more important in regulating free intracellular Ca^2+^ during muscle contraction and relaxation. Future studies that examine whether genetic deletion of *Gap43* alters fibre type distribution and fatigue resistance, similar to that conducted with *Nrip*-null mice [9], will provide more insight towards the role of GAP43 in regulating CaM activation of CaN and CaMKII. In addition, given the importance of Ca^2+^ regulation and CaM signalling in cardiomyocytes, investigations focusing on the potential role of GAP43 in cardiac muscle will be of interest.

## 5. Physiological Significance

**Figure 1:**
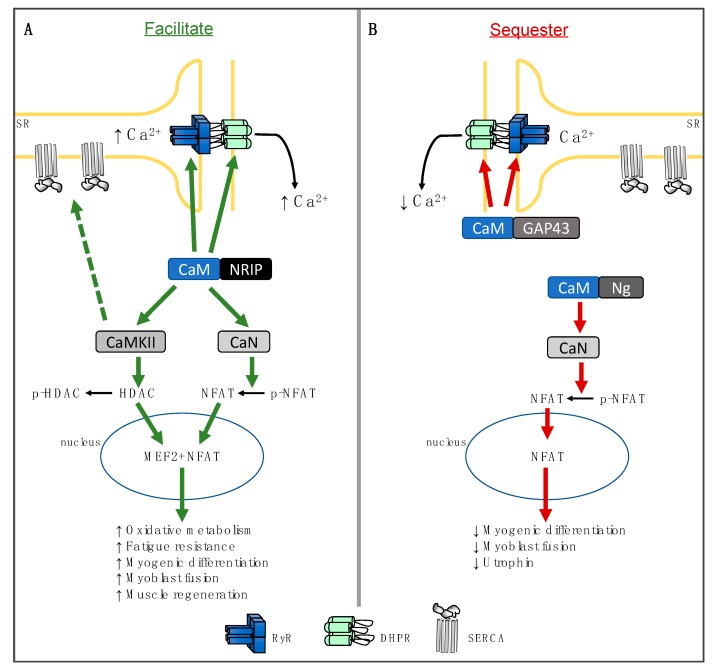
Simplified scheme of the physiological roles of calmodulin (CaM)-binding proteins, nuclear receptor interacting protein (NRIP), neurogranin (Ng), and growth-associated protein 43 (GAP43) in skeletal muscle. (**A**) CaM-binding proteins that facilitate CaM binding with its downstream targets leads to their subsequent activation (green arrows). Recent evidence has revealed a role for NRIP in facilitating CaM binding to ryanodine receptor (RyR) and dihydropyridine receptor (DHPR), thereby contributing to increased sarcoplasmic reticulum (SR) Ca^2+^ release [9]. NRIP also facilitates CaM binding and activation of CaMKII and CaN. Activated CaMKII phosphorylates class-II histone deacetylases (HDACs), thereby activating myocyte enhancer factor 2 (MEF2). CaN dephosphorylates nuclear factor of activated T-cell (NFAT), leading to its nuclear entry. Together MEF2 and NFAT increase the expression of genes associated with oxidative fibre type, fatigability, myogenic differentiation, myoblast fusion, and muscle regeneration [9]. The dashed green arrow illustrates the hypothetical scenario by which enhanced CaM-dependent kinase II (CaMKII) activation with NRIP can lead to increased SERCA-mediated Ca^2+^ uptake, thereby enhancing total SR Ca^2+^ potentially by phosphorylating phospholamban (not shown here) and relieving its inhibition of SERCA. (**B**) CaM binding proteins that sequester CaM away from its downstream targets lead to a subsequent reduction in their activation (red arrows). Ng binds to CaM and prevents its binding to CaN, leading to a reduction in myogenic differentiation, myoblast fusion, and utrophin expression [28]. GAP43 binds to CaM and prevents it from binding to RyR and DHPR, thereby reducing SR Ca^2+^ release. The role of NRIP in maintaining normal cardiac function [11] is not shown here.

By uncovering the role of these endogenously expressed proteins that either facilitate CaM binding with its downstream targets (NRIP, Figure 1A) or sequester it away from its downstream targets (Ng and GAP43, Figure 1B), a new area of research has emerged in the field of muscle physiology. With the abundance of experimental support demonstrating the physiological importance of CaM-dependent and CaM-regulated proteins (i.e., CaN, CaMKII, RyR, and DHPR) in muscle, we can anticipate that these CaM-binding proteins, and those yet to be uncovered and characterized, could have several physiological implications.

For example, previous research has shown that activating CaN in rodent skeletal muscle via overexpression of its constitutively active form promotes the slow-oxidative phenotype, leading to a reduction in fatigability [46]. Genetic deletion of *calsarcin-2*, an endogenously expressed CaN inhibitor particularly enriched in fast-twitch glycolytic fibres, also leads to a shift toward slow-oxidative fibres and a concomitant increase in fatigue resistance [47]. Alternatively, inhibiting calcineurin signalling pharmacologically with cyclosporine or FK506 treatment [6], or by overexpressing its endogenously expressed inhibitor, regulator of calcineurin 1 (RCAN1) [48], promotes a switch toward the fast-glycolytic fibre type, leading to an enhancement in fatigability. Thus, altering CaN activation upstream via CaM regulation could have important implications in muscle fibre type composition and endurance. Indeed, the binding of NRIP to CaM facilitates CaM/CaN and CaM/CaMKII activation, leading to a switch toward the slow-oxidative fibre type and fatigue resistance [9]. However, the roles of CaM sequestering proteins (Ng and GAP43) on muscle fibre type composition and endurance have not yet been examined. Further, both CaN and CaMKII are activated with chronic endurance training and contribute to the benefits associated with regular exercise (i.e., mitochondrial biogenesis) [49,50]. Studying whether these CaM regulatory proteins potentiate or reduce the benefits associated with chronic endurance training would have physiological implications for exercise performance and health.

It is known that the type I fibres are more insulin sensitive and are better at oxidizing fat compared to their type II glycolytic counterparts. Transgenic overexpression of the constitutively active form of CaN in mice improves insulin-stimulated glucose uptake, enhances fatty acid oxidation, and protects against high fat diet-induced glucose intolerance [51]. Recent evidence also implicates CaN in the activation of whole-body thermogenesis [52]. Genetic deletion of *Rcan1* led to a significant increase in muscle sarcolipin (SLN) expression, which mediates muscle-based thermogenesis by uncoupling SERCA-catalyzed Ca^2+^ transport [53,54]. *Rcan1*-null mice also had increased expression of uncoupling protein 1 (UCP-1) in adipose tissue, which is a mitochondrial uncoupler well-known to have implications for diet-induced obesity [55,56]. In turn, when *Rcan1*-null were fed a high fat diet, they experienced less weight and were more glucose tolerant when compared with WT mice [52]. Therefore, it will be of interest to determine whether regulating CaN signalling via upstream control of CaM availability would have a similar impact on diet-induced obesity and glucose handling as those observed with other endogenous regulators of CaN, such as RCAN1.

Duchenne muscular dystrophy (DMD) is a severe X-linked muscle-wasting condition caused by an absence of the functional dystrophin protein that primarily affects the fast-glycolytic fibres [57,58]. This is partly because oxidative fibres express more utrophin—a dystrophin homolog [41]. The importance of regulating CaM and its downstream activation of CaN and CaMKII have been made evident with *mdx* mice (DMD mouse model) overexpressing a synthetic CaM binding protein that sequesters CaM and limits its availability [59]. These *mdx* mice exhibited impairments in both CaN and CaMKII signalling, and not surprisingly, the CaMBP overexpressing *mdx* muscles displayed a worsened dystrophic pathology with reductions in utrophin expression [60]. In addition to its effects on utrophin, CaN’s activation of muscle regeneration is also important for muscular dystrophy [61]. When CaN signalling was blocked with cyclosporine A, *mdx* muscles had fewer centrally nucleated fibres (marker of regeneration), more endomysial fibrosis and mononuclear cell infiltration, and were 30%–35% weaker compared with the vehicle control [62]. SLN also functions as a CaN activator in muscle in addition to uncoupling the SERCA pump [63]. Genetic deletion of *Sln* in *mdx* mice led to impairments in CaN signalling, thereby reducing utrophin and stabilin-2 expression and exacerbating muscle weakness in *mdx* mice [64]. Collectively, these studies demonstrate the importance of CaN and CaMKII in mitigating a dystrophic pathology. Indeed, there are other muscle diseases in which activating these CaM-dependent proteins could lead to physiological benefits, including myotonic dystrophy type 1 [65] and centronuclear myopathy [66]. Therefore, the regulation of CaM availability will have a significant physiological impact on a number of muscle myopathies.

There is also some evidence suggesting that CaN may promote skeletal muscle hypertrophy; however, there is considerable discrepancy [67]. Chronic administration of cyclosporine A or FK506 in mice inhibits CaN activation while preventing the fast-to-slow fiber type transformation that occurs during functional overload of the plantaris muscle [68]. In some cases, this has also been shown to prevent the increase in muscle mass and individual fiber cross-sectional area commonly observed in the overloaded plantaris [69,70]. However, there have also been studies that have reported no such effect of CaN on muscle mass (for a review see Reference [67]). Indeed, the role of CaN in stimulating muscle hypertrophy is complicated due to the fact that CaN also promotes the slow-oxidative fibre type that is inherently smaller than fast-glycolytic fibres. Nonetheless, CaN is well-known to play a role in myoblast fusion [27,29], which could enhance muscle regeneration and myofiber size [71,72].

In contrast with skeletal muscle, it is well known that CaN contributes to pathological but not physiological cardiac hypertrophy [73,74]. For example, mice lacking calsarcin-1, an endogenous calcineurin inhibitor found specifically in slow-oxidative fibres, had an augmented hypertrophic response to thoracic aortic banding, but not in response to exercise [74]. These findings raise a potential dichotomy, whereby activating CaN may benefit skeletal muscle performance and regeneration but may lead to pathological cardiac hypertrophy. In this respect, NRIP has been shown to activate CaN and enhance fatigue resistance in skeletal muscle [9]; however, NRIP was not shown to have a role in CaN signalling in cardiac muscle [11]. In fact, genetic deletion of *Nrip* in cardiac muscle led to muscle weakness, cardiac hypertrophy, and ventricle dilation [11]. Further, CaN activation has been shown to protect against dilated cardiomyopathy [75]. Together these findings highlight the importance of examining the potential physiological roles of these endogenously expressed CaM-binding proteins in both cardiac and skeletal muscle.

## 6. Conclusions

Though only recently emerging as a field of study, CaM regulation via endogenously expressed IQ motif proteins, such as NRIP, GAP43 and Ng, may have important implications for skeletal and cardiac muscle health. We anticipate that future studies of CaM regulation will uncover novel mediators/regulators of CaN and CaMKII signalling and intracellular Ca^2+^ dynamics, which could potentially be exploited to enhance muscle performance and treat conditions such as obesity, type 2 diabetes, and muscular dystrophy. However, a potential dichotomy may exist when activating CaN in skeletal muscle vs. cardiac muscle, where pathological hypertrophy in the latter could occur. Thus, it is important to investigate the effects of these CaM-binding proteins in both skeletal and cardiac muscle.

## Abbreviations

AID	Autoinhibitory domain
CaM	Calmodulin
CaMKII	Calmodulin dependent kinase II
CsA	Cyclosporine A
CNM	Centronuclear myopathy
CaMBP	Calmodulin-binding protein
DHPR	Dihydropyridine receptors
DMD	Duchenne muscular dystrophy
DM1	Myotonic dystrophy type 1
GAP43	Neuron-growth-associated protein 43
MEF2	Myocyte enhancer factor 2
NFAT	Nuclear factor of activated T-cells
NRIP	Nuclear receptor interacting protein
Ng	Neurogranin
PGC1-	Peroxisome proliferator-activated receptor gamma coactivator 1-alpha
PLN	Phospholamban
RyR	Ryanodine receptor
SR	Sarcoplasmic reticulum
ROS	Reactive oxygen species
RCAN1	Regulator of calcineurin1
SERCA	Sarco(endo)plasmic reticulum Ca^2+^ ATPase
SLN	Sarcolipin
UCP1	Uncoupling protein 1
